# On the State and Condition of Lunacy in Ireland
*Report of the Commissioners of Inquiry into the State of the Lunatic Asylums, and other Institutions for the Custody and Treatment of the Insane in Ireland.—(*Blue Book*.) 1858.Observations on the Report of the Commissioners of Inquiry into Lunatic Asylums, &c. (Ireland), in a Letter to the Right Honourable Lord Viscount Naas, M.P., Chief Secretary. By J. Nugent, M.D., Inspector of Lunatic Asylums. (Her Majesty's Stationery Office.) 1858.


**Published:** 1859-01-01

**Authors:** 


### Art. VII.—ON THE STATE AND CONDITION OF LUNACY IN IRELAND.*

In 1856 a Royal Commission was appointed to inquire into the state of Lunatic Asylums and other Institutions for the custody and treatment of the Insane in Ireland, and into the present state of the Law respecting Lunatics and Lunatic Asylums in that part of the United Kingdom. The Report of the Commissioners has now been made public, and it is unfavourable, not only as regards the condition and management of both public and private institutions for the insane in Ireland, but also as regards the existing state of the Irish Law of Lunacy. From several omissions, however, and also from not a few misapprehensions of the Commissioners, it appears to us that the unfavourable aspect of their Report has been unnecessarily, although, without doubt, unintentionally, heightened, and that,

* Report of the Commissioners of Inquiry into the State of the Lunatic Asylums, and other Institutions for the Custody and Treatment of the Insane in Ireland.—(*Blue Book.*) 1858.

Observations on the Report of the Commissioners of Inquiry into Lunatic Asylums, &c. (Ireland), in a Letter to the Right Honourable Lord Viscount Naas, M.P., Chief Secretary. By J. Nugent, M.D., Inspector of Lunatic Asylums. (Her Majesty's Stationery Office.) 1858.

in consequence, the Report does not present a correct notion of the actual state and condition of Lunacy Legislation and Management in Ireland.

The returns laid before the Commission show that, on the 1st of January, 1857, the number of insane poor in Ireland amounted to 9286, of whom 5934 were maintained at the public charge, and 3352 were at large. The returns made to the Commissioners do not correspond with the returns of the Inspectors of Lunacy for the same period. These returns give the total number of insane in Ireland, exclusive of epileptics at large and in workhouses, as being 11,452, of whom 5441 were at large. The inspectors estimate that, of the total number of insane at large, as recorded in their returns, 600 lunatics and idiots were not paupers. Thus, according to this calculation, the insane poor would amount to 4841. The returns upon which the Commissioners' enumeration is founded distinctly specified that the "poor" alone should be included; the inspectors' estimates were founded on returns obtained irrespective of social condition.

The Census returns show that the total number of insane in Ireland on the 31st of March, 1851, was 9980. A comparison of the Census and of the Lunacy Inspectors' returns, "made at an interval of between five and six years, and obtained through the same sources of information, shows a considerable increase in the amount of insanity in Ireland."—(p. 2.)

It is to be regretted that the Commissioners have not given an estimate of the proportion of insane to population in each of the two periods referred to, and also of the probable rate of increase in the amount of insanity.

The insane poor, who are maintained at the public charge, are distributed in the District Asylums (sixteen in number), the workhouses, the gaols, and the Central Criminal Asylum.

Concerning the District Asylums, the Commissioners report that the rules which exist (themselves imperfectly conceived) for the government of those institutions are frequently imperfectly carried out, disregarded, or altogether violated; that the books which are required to be kept for recording, at regular periods, the condition of the inmates, are either not kept, or if kept, there is a want of uniformity in the mode in which the entries are made; that the Governing Boards of the institutions do not, as a rule, meet sufficiently often; that there is great diversity in the rules of admission; that the hygienic arrangements of both the old and the new asylums are not satisfactory, particularly the ventilation and warming; that in several asylums there is an inattention to cleanliness; that the provision for the recreation of the inmates is deficient, if not altogether wanting; that the wards have commonly a bare and uncomfortable aspect; that, though the dietary usually appeared equal, if not superior, to that which the inmates had been previously accustomed to, there were great variations in different asylums, and the returns of consumption showed inexplicable inconsistencies with the tables of allowances; and the Commissioners remark, "If the returns of consumption are correct, the patients cannot receive the amount of food professed to be allowed to them."—(p. 14.) Further, the Commissioners object to the use of skimmed-milk and butter-milk in asylums. The Commissioners report, also, that the bedding is ample in quantity, but not sufficiently good in quality; that the bedsteads are often of bad construction; that the clothing is in general sufficient, and of fair quality, but that it requires improvement; and that the distribution of out-door exercise, or labour, among the inmates, is not satisfactory on the whole. The Commissioners also object to the treatment of the sick in cells, as is practised in several asylums, even where a proper infirmary exists—which, however, in a few instances, was found to have been converted to another use. Moreover, the Commissioners report that the prescription, wine, and dietary books are negligently kept, and that there is a "culpable disregard" of the rule which regulates the use of restraint. This rule requires that "the Manager is to take charge of the instruments of restraint, and is not, under any pretence, to allow the unauthorized use of them to any person within the establishment; all cases placed under restraint, seclusion, or other deviation from the ordinary treatment, are to be carefully recorded by him in the daily report, with the particular nature of the restraint or deviation resorted to; but in no case shall the shower-bath be resorted to without the authority of the physician."—(p. 16.) In some instances the Commissioners found that the managers were not even aware of this rule; in others, it was found that the instruments of restraint were left in charge of the attendants; and several instances are mentioned in the Report in which restraint had been had recourse to without the knowledge either of the manager or visiting physician of the asylum. In two of these instances the restraint was unjustifiably harsh and protracted.

It is a somewhat singular fact that, while the Commissioners have, throughout their Report, kept constantly in view the great proposition that an asylum should be an institution for the treatment as well as for the detention of the insane, they should have altogether passed over, without observation, the data submitted to them, and printed by them in the Appendix to the Report, which indicate the character of the asylums of Ireland as curative institutions for the insane. When these data, so important to a right knowledge of the subject of investigation, are examined, a suspicion is excited that the generally unfavourable opinion of the Commissioners upon the District Asylums arises in no small degree from their having raised a false standard of comparison. We do not desire for a moment to convey an impression that we in any way approve, or would wish the continuance of those irregularities and defects in management that the Commissioners report; but the omission upon their part of any allusion to the most creditable, as well as most important feature of the asylum returns, is a circumstance much to be regretted.

From the Tables of Admissions and Discharges of the different District Asylums, it would appear that the proportion of recoveries, calculated on the admissions, for the whole of the District Asylums, on an average of five years (1852-56), was 39·2 per cent.; and that the mortality of the same asylums, calculated upon the whole number of insane within them during the same period, was 10·5 per cent. These proportions, both of recoveries and deaths, compare very favourably with those occurring in English County Asylums, the mortality being below, and the recoveries almost equal to the proportions found in those institutions. Drs. Bucknell and Tuke give the following averages (for periods not stated) of the recoveries and mortality of fifteen English County Asylums:—

| | Proportion of Recoveries per cent. of admissions. | | Mean Annual Mortality per cent. Resident. |
|---|---|---|---|
| Average of six English County Asylums receiving private and pauper patients . . . | 46·87 | — | 10·46 |
| Average of nine English County Asylums receiving paupers only . . . . . . . . | 36·95 | — | 13·88 |
| Average of the fifteen Asylums | 41·91 | — | 12·17 |

The general condemnation of the Commissioners is, so far as we can gather from the medical returns with which they furnish us, and from which the foregoing statistics of recoveries and mortality in the District Asylums are taken, in a great measure inconsistent with the medical results obtained in those institutions. Faults there undoubtedly are, both in the hygienic condition and general management of the District Asylums; but we must not, on that account, ignore their merits.

Moreover, Dr. Nugent, one of the Inspectors of Lunacy in Ireland, in a letter to the Right Honourable Viscount Naas, M.P., Chief Secretary, points out that the opinions of the Commissioners respecting the dietary of lunatics, the disuse of the infirmaries in several asylums, and the negligent use of the prescription and wine books, are founded on misapprehensions. The inconsistencies in the consumption and the diet-tables, to which the Commissioners refer, may be explained by the variations ordered from the ordinary diet by the physicians. The disuse of the infirmaries in several asylums can be, and is, satisfactorily accounted for; and it appears that a bye-law exists (however ill-advised it may be) giving the physician the option in using the books referred to.

Dr. Nugent seeks also to explain in some degree the instances of cruel restraint mentioned by the Commissioners. When, however, as would appear to be the case in the District Asylums, the number of attendants is not sufficient for the effective carrying out of a system of non-restraint, and the wages paid to the attendants are not liberal enough to secure persons of a degree of intelligence that would enable them to comprehend and carry out fully the requirements of a non-restraint system, it becomes almost necessary to use more or less restraint in the treatment of the insane; but the liability to abuse becomes also great, and the necessity for stringent rules regulating the use of the means of restraint, and for the strict observance of those rules, cannot be too carefully enforced.

Concerning the lunatic wards in workhouses, the Commissioners report that they are most unsuitable for the detention of the insane; and upon the condition of the insane in them at the present time the Commissioners write:—

"The unfortunate creatures have commonly no one to attend to them but some of the other pauper inmates, who are but little capable, or little inclined to concern themselves with looking to their wants or necessities. The result is, that the condition of these wards, and the lunatics detained therein, is usually most unsatisfactory. In many cases the bedding is ill attended to, the persons of the lunatics were often most filthy, their clothing bad, and no effort at cleanliness was observable, in this apparently condemned division of the workhouse. In some workhouses, however, the bodily condition of these afflicted beings was as carefully attended to as in an asylum, though we cannot say that in any their moral treatment was much studied. The cases, are, however, generally chronic or idiotic."—(p. 18.)

Dr. Nugent directs attention to a fact, upon which the defective care of lunatics in workhouses mainly depends, and which seems to have escaped the attention of the Commissioners, namely, that the strict law of Union discipline does not recognise paid servants in workhouses, and forbids their employment, "consequently the demented and idiotic classes are dependent on the *charitable* offices of the pauper inmates of these institutions."—(*Letter*, p. 11.)

Concerning the gaols as places of reception for lunatics, the

Commissioners report that they are most unfitted for such a purpose, from the absence of all arrangements in these buildings for the treatment of the insane; and concerning the Central Criminal Asylum, which was opened for the reception of criminal lunatics in 1850, the Commissioners report that, although it is provided with all modern improvements, and well conducted, the arrangements for ventilation and heating seem to be defective, and there is a general absence of any means of amusement for the inmates, and of any pictures or decorations to relieve the monotony of the whitened walls of the day-rooms and galleries.

Of the condition of other establishments for the insane, according to the Report, the following is a summary:—

Previous to 1857, an establishment, connected with the House of Industry of the city of Dublin, and known as the Hardwicke Cells, existed in that city, and afforded accommodation for 108 lunatics. This institution was very inefficiently managed, and in 1857 the inmates were removed to a new establishment at Lucan. Of the character and management of this establishment, which is maintained by a Parliamentary grant, the Commissioners speak in favourable terms.

Concerning the Private Asylums, the Commissioners report that they, "generally, are not well adapted for their purpose. They are usually old mansions, or private houses, which have been converted to their present use, and their arrangements are not convenient for the purpose of an asylum" (p. 32); and concerning the benevolent institutions for the insane, two in number—St. Patrick's Hospital, Dublin, and the Bloomfield Retreat—the Commissioners report that the objects of the former institution have not been satisfactorily carried out, the reception of paying patients having become the most marked characteristic of what was intended to be a charitable institution; but of the Bloomfield Retreat, a small asylum belonging to the Society of Friends, the Commissioners express approbation.

Of the condition of the insane poor who are at large in Ireland, the Commissioners give the following classified summary:—

| Insane. | Idiotic. | Well treated. | Neglected. | Dangerous. | Troublesome. | Harmless. | Resident with Relatives. | Resident with other persons. | Living alone. | Vagrant. |
|---|---|---|---|---|---|---|---|---|---|---|
| 691 | 2661 | 1767 | 1585 | 62 | 261 | 3029 | 2371 | 336 | 80 | 565 |
| 3352 | | 3352 | | 3352 | | | 3352 | | | |

The asylum accommodation, notwithstanding that this has been considerably extended within a few years, is still inadequate. The insane poor at large, according to the Commissioners' returns, amount, as previously stated, to 3352, and Dr. Nugent considers that additional accommodation ought to be provided for 2400, that being about the number of insane poor at large, who, according to his calculations, require to be placed under proper control. In several districts the want of additional accommodation is severely felt; and if several of the recommendations of the Commissioners are carried out, the necessity for further accommodation will be still more felt.

A defective state of the law respecting lunatics and lunatic asylums is to be regarded, according to the Commissioners, as the main source of the imperfections which they report in the condition and management of lunatic asylums generally in Ireland. Moreover the Commissioners imply that no sufficient means exist by which the perfect action of the law, even as it at present stands, could be effectively secured. The Inspectors of Lunacy, with whom rest the superintendence and direction of asylums, have so great an amount of duty to perform, that it is impracticable for them to give that narrow attention to the internal regulation of asylums which might be desired, or is necessary; and the committal of all that relates to the building, fitting up, and extension of asylums, to the present Board of Control, as well as the mode in which the power is evoked, that rests with the Lord Lieutenant in Council, to erect new asylums, are not the means best fitted to obtain good results.

An entire reconstruction of the lunacy laws is proposed by the Commissioners, and they suggest that a Central Board should be created which should have the full direction and superintendence of all asylums, public and private, as well as the control of single lunatics. This Board, it is further suggested, should consist of three salaried members, two of whom should be members of the medical and one of the legal profession. The Commissioners do not propose to associate any unpaid Commissioner (as in the case of England and Scotland) with the salaried Commissioners, for the following reasons:—"First, because we do not think that any unpaid members, whose services could be made available for the purpose, would give additional weight to the authority of the Board; secondly, because it is not likely a regular attendance would really be ensured on the part of the persons so appointed, and whose position and ability might point them out as qualified for the discharge of the duties belonging to the Board; lastly, because we think it of great importance that the Commissioners should not merely sit at the Central Office, to order and direct the management of the asylums, but that they should be themselves kept constantly informed of the actual condition of the asylums, and the lunatics under their control, by discharging the duties of inspection" (p. 26). The Commissioners should perform the duties of inspectors, and should have full power of examination into all that relates to the management and condition of asylums, with the right of entrance into them at any hour, night or day; they should, also, have the right to sit at the meetings of Boards of Governors, but not to vote. In all that relates to the increase of asylum accommodation and the erection of new asylums, the Central Board would advise the Government, and superintend the carrying out of such measures as might be determined upon; the Board would, also, have supreme control over the licensing and government of private asylums, and it would become the medium of simplifying and making more satisfactory, the proceedings respecting Chancery lunatics.

At present rate-payers or their representatives, Grand Juries and Town Councils, have no voice whatever in determining the management of asylums, the increase of asylum accommodation, and the erection of new asylums. The two latter measures rest altogether with the Executive Government, which also provides the funds necessary for the construction, alteration, and expenditure of asylums, recovering the sums advanced by rates laid upon the district for which the asylum is erected. The Commissioners propose that the rate-payers, or their representatives, should in future have a voice in a matter which so nearly concerns their interest as the provision of additional asylum accommodation, or the erection of new asylums; and that Grand Juries and Town Councils should have the privilege of electing a portion of the governors of asylums, now nominated entirely by the Government.

The Commissioners also suggest that a new code of regulations should be drawn up for the internal management of public asylums.

We concur in the suggestion of the Commissioners in regard to the formation of a Central Board having the superintendence and direction of the general and internal management of lunatic asylums and the control of single lunatics, but we doubt whether the constitution of the Board, as proposed by the Commissioners is that best calculated to secure its objects; and whether, if the suggestions were carried out concerning the relations which should exist between the Central Board, Boards of Governors and Grand Juries, unless the opinions of the two latter bodies are to have more weight than seems to be contemplated by the Commissioners, the relations proposed would not lead to frequent and perplexing embroilments.

Dr. Nugent makes a suggestion respecting the constitution of the proposed Central Board, which merits attention, and with which, if it be feasible, we are disposed to coincide. He writes—

"Though I may be opposed to the constitution of a Board of three —two physicians and a lawyer—one on a larger and more influential basis may be worthy of much consideration by your lordship. I allude to a Central Board, of which the Lord Chancellor would be Chairman, with four unpaid Commissioners, one of whom might be a Judge of the Court of Queen's Bench (for criminal lunacy), and two others members of the Privy Council, with three paid Commissioners, two of them to be physicians, the third a barrister. A Board so constituted must, *per se*, command full control, and an unquestionable authority throughout the kingdom."—(*Letter*, p. 18.)

We may not dwell upon the numerous suggestions which are made by the Commissioners respecting the particular management of asylums and alterations of the law, except in one instance relating to medical arrangements. The Commissioners agreed in suggesting that a Resident and Visiting Physician should be attached to each public asylum, and four of their number considered "that the resident physician should be solely responsible for the treatment of the patients, both as regards their bodily health and their mental disease; but that he should be assisted, when necessary, by a visiting physician, whose duties, however, should be confined to cases where his attendance may be required in consultation with the resident physician."—p. 9. One of the Commissioners (Dr. Corrigan) considers, however, that the attendance of the visiting physician—

"Should not be dependent on the discretion either of the Resident Physician or Local Board, but that he should visit the institution daily, that while the Resident Physician and Manager should have general charge of the institution, and be responsible for the treatment of the insane, as such, the duty of the Visiting Medical Officer in this regard should extend only to cases where his attendance may be required in consultation by the Resident Physician, but that the Visiting Physician should daily visit all cases whatever confined to bed, in seclusion, or under restraint; that he should see, with as little delay as possible all cases of injury, accident, and child-birth, and record such observations on them as may appear requisite; that he should be directly responsible for the treatment of the sick as distinguished from the mere insane; that, in all cases of discharged patients, the certificate of discharge should be signed by both Resident and Visiting Physicians; and that, in all cases of death, the record of the illness and cause of death should be signed by both the Resident and Visiting Physicians."—(p. 9.)

It is not apparent from the Report upon what grounds Dr. Corrigan forms his opinion that the medical functions of the resident and visiting physicians should differ, or why indisposition occurring in an insane person apart from and in addition to his insanity, should constitute a sufficient reason for his being transferred to the charge of another medical man, who is not responsible for the treatment of the mental affection. That a patient should be seen daily by two medical men, the one of whom is to confine his attention and to be solely responsible for his mental state, the other for his bodily state, seems to us a speciality of practice which, to say the least, is somewhat paradoxical; and we cannot conceive what advantage either the patient or the institution can derive from it. Moreover, that the visiting physician, who is to be solely responsible for the bodily ailments of the lunatics, should also act as consulting physician to their mental ailments, makes the paradox, if we may so write, still more paradoxical.

Dr. Nugent, although appearing to coincide with Dr. Corrigan's opinion concerning the duties of the visiting and resident physicians, differs from the opinion of the Commissioners, that the resident physicians should not perform civil duties; for he writes—"I really do not see (if aided by a Visiting or Consulting Physician) what employment the Resident can have to occupy his whole time as a public salaried officer [particularly if the patients do not exceed 200 or 250], unless he superintends the general domestic economy of the establishment."—(p. 5.)

The Commissioners direct attention to the insufficiency of the wages of the domestics in the public asylums, and the inadvisability of low wages under these circumstances. We may add that the salaries of the officials appear to be on a scale which is not commensurate with the importance of the duties performed. In particular, the salaries of the inspectors of lunacy are characterized by a parsimony which contrasts very strongly and unsatisfactorily with the salaries attached to some of the legal appointments of secondary importance in connexion with the Irish government.

We have only entered so far into the details of the Commissioners' Report as would enable us to present to our readers an idea of its general tendency and character. Dr. Nugent, in the letter to which we have several times referred, has officially and most justly protested against the almost unbroken series of condemnatory conclusions of the Commissioners respecting the management and condition of the lunatic asylums in Ireland. From his long and intimate acquaintance with everything that relates to these institutions, Dr. Nugent's opinions merit very high consideration; and with regard to the important questions of increased asylum accommodation, district requirements, expenditure, and monetary arrangements, the value of his views and suggestions cannot be over estimated.

In addition to the very creditable character of the asylums in Ireland, as curative establishments (to which we have already referred), Dr. Nugent states that they have been remarkably free from abuse, and that accidents are of rare occurrence in them, which he attributes to the carefulness of the attendants. "Only four cases of suicide, and not one of a homicidal nature, are recorded as having taken place within five years, notwithstanding that in that period no less than 2000 lunatics, committed to gaols on sworn depositions as 'dangerous' to themselves or others, had been transferred thither; and that at the date of their (the Commissioners') statistical returns, there were 551 of the class still remaining."—(*Letter*, p. 2.)

---

